# The Effect of Eating Habits’ Quality on Scholastic Performance in Turkish Adolescents

**DOI:** 10.3390/bs10010031

**Published:** 2020-01-10

**Authors:** Aleksandra S. Kristo, Büşra Gültekin, Merve Öztağ, Angelos K. Sikalidis

**Affiliations:** 1 Department of Food Science and Nutrition, California Polytechnic State University, San Luis Obispo, CA 93407, USA; akristo@calpoly.edu; 2 Department of Nutrition and Dietetics, Istanbul Yeni Yuzyil University, 34010 Istanbul, Turkey; busra-gultekin@windowslive.com (B.G.); merve.oztag@yeniyuzyil.edu.tr (M.Ö.)

**Keywords:** adolescent students, eating habits, healthy eating, scholastic success, Turkey

## Abstract

Evidence associates scholastic performance to quality of eating habits. However, there is limited information on this topic in Turkey, an emerging economy with notable disparities. Our work aimed to evaluate the effect of eating habits quality of high-school students in Turkey, on the Scholastic Aptitude Standardized Examination (TEOG) scores. The study was conducted in 29 different cities in Turkey during the academic year 2016–2017, involving students of ages 14–17 years (up to senior-high school). A dietary habits survey developed and validated for this population was distributed over the internet in February 2017. Apart from students’ TEOG scores, Family Affluence Score (FAS) was used to categorize the students into low, medium, and high financial standing. Eating Habits Score (EHS) was calculated by using a validated scoring system. A working sample of 298 participants was used. Based on our results, we observed that there is a significant positive correlation between EHS, FAS and success rate of students as assessed by TEOG scores. Further research on this subject should be conducted in combination with intervention studies to reveal potential strategies and policies that would enhance positive behavior change as it relates to nutritional habits, aiming at improved scholastic performance and overall health throughout lifespan.

## 1. Introduction

Cognitive and academic performance are significantly affected by numerous determinants, including school quality indicators (i.e., facilities, teaching quality, allocated teaching time), of family characteristics (i.e., socioeconomic status (SES), parents’ educational level, and attitudes towards education). Other determinants further include more of individual characteristics (i.e., natural inclination, aptitude, motivation, and behavior) all of which have established interdependent effects [1,2,3]. While dietary patterns can affect cognitive ability and behavior in adolescents, nutrient composition and meal pattern can over-time exert long-term, beneficial or adverse effects as these may relate to cognition [1,4,5]. Breakfast as part of a healthful diet and lifestyle, can positively impact children′s health and overall well-being, especially including high-fiber and nutrient-rich whole grains, fruits, and dairy products [4]. Therefore, it can be argued, within the aforementioned context, that an individual’s health and nutritional status are additional determinants of interest and indicators/predictors of scholastic performance [5].

Nutrition and optimal diet are key developmental drivers regarding ideal development both at the physical as well as the mental and cognitive level in adolescents. In this context, an improved cognitive and mental state, translate into higher potential for improved scholastic performance subsequently leading to academic success, hence empowering students with more knowledge and superior set of skills, which in turn can lead to the attainment of higher socioeconomic status. From a societal perspective, such developments also translate into added per capita value for the economy, thus leading to a more vibrant workforce and robust overall economic growth. The dietary choices and patterns observed in adolescent students have been associated with outcomes related to scholastic performance and overall academic success [6,7,8].

Nutritional deficiencies during critical periods of brain development can confer a lasting impact on intellectual development. This becomes particularly of concern in developing countries, or emerging economies at epidemiological transition, where significant disparities and inequalities are observed, and are in part reflected by poor dietary status and/or nutrient deficiencies. This can become a sizable problem with clear repercussions both for the individual and the economic prosperity of an entire country. While in developing countries there is overwhelming evidence that the adequacy of diet, particularly while the brain is rapidly growing/developing, has significant and lasting implications for cognitive functioning, in industrialized countries however, there is little while sometimes even conflicting evidence to confidently draw relevant conclusions. There are however, well-designed studies which seem to support the principle that relatively minor differences in the diet can potentially be of measurable influence regardless of the setting [8].

More specifically, inadequate and/or unbalanced nutrition have been reported to shorten attention span, reduce perception, cause difficulty in learning, induce behavioral disorders and absenteeism while reduce overall scholastic success [8]. Studies indicate that regularity in meal consumption, particularly pertinent to breakfast, is positively associated with academic achievement [8]. Students taking breakfast regularly, on an average, typically demonstrate higher levels of concentration as well as greater ability towards comprehension, memory and problem solving. Interestingly, while breakfast is considered beneficial for cognitive and academic performance in school children, it also tends to be the most frequently skipped meal, especially among adolescents [6].

The quality of meals has also been shown to affect certain aspects of scholastic performance. More specifically, it has been reported that intelligence quality (IQ) test scores and memory test scores are higher for children who consume more fruits, vegetables and home-cooked meals, and also higher for children who consume sufficient micronutrient amounts (folate, iron, B vitamin complex, omega 3 fatty acids, polyunsaturated fatty acids) compared to children who do so less. On the contrary, high consumption of sugary foods, salty snacks, fast food and/or inadequate consumption of dairy products and nutrient-dense foods (vegetables, fruits, meat, fish, eggs) has been found to be associated with poor overall scholastic performance in some settings [9,10].

Suboptimal nutrition and hence generation of inadequate learning opportunities are both factors inversely linked to SES, and pose significant risks for poor overall health, wellbeing and achievements [11]. Notably, SES refers to a complex, multidimensional construct that reflects financial resources and capital, while the most common indicators/determinants of SES (as SES pertains to adolescents) constitute: household income, parental education, and parental occupation [11]. Hence, there is a strong link between SES, health and level of education attainment. Furthermore, nutrition can function as a mediator via which SES impacts health and scholastic success. Scholastic success and relevant implications aside, optimal nutritional habits acquired in the young age are positively impacting individuals for the rest of their life, arguably contributing to better health and lower disease risk. This is an additional reason as per the importance of studying dietary and/or lifestyle habits in the adolescent population and aim to identify strategies and schemes for optimizing these behaviors, thus maximizing benefits to individuals and society, while also inform best practices and policies.

Turkey is a country in epidemiological transition still with significant inequalities as well as health disparities. The country is concurrently facing challenges of over-nutrition and poor dietary habits associated with rapid adoption of Western dietary patterns similar to those seen in China and India [7]. Simultaneously, there are significant segments of the general population in poor dietary conditions due to food insecurity and/or limited food access due to primarily financial reasons [7]. Furthermore, there is a significant lack of available data pertaining to how nutrition and diet relate to scholastic success of students in the modern Turkish food and nutrition landscape, so while important, this is an understudied topic. Notably, there is significant variation in terms of bodyweight as, a significant portion of the adolescent population faces problem of overweight and obesity or underweight. Moreover, anemia and nutrient, particularly micronutrient deficiencies, are quite prevalent [7]. These are observations typical of countries in epidemiological transition. In Turkey, nutritional education is limited and of controversial effectiveness since there are deficits as per practical components such as home-economics cooking lessons, with inconsistency in the overall curriculum. Furthermore, there is a limited number of trained nutritionists/dietitians in the country considering the overall population due to limited and only recently created higher education programs producing nutrition graduates [7]. In this sense, Turkey demonstrates a series of interesting characteristics from a Nutritional Epidemiology and Public Health perspectives. In this study, we aimed to assess the relationship between adolescent students′ eating habits and their success in several school settings in different cities in Turkey. We hypothesized that nutrition habits do affect students’ scholastic performance as assessed by standardized test scores, in a fashion proportional to students’ achieved eating habits score.

## 2. Materials and Methods

### 2.1. Study Design, Participating Population and Characteristics

This was a cross-sectional analytical pilot study aiming to examine the relationship between Turkish adolescent students’ eating habits (independent variable) as measured by eating habits score (EHS) and their scholastic success as measured by standardized scholastic aptitude tests (TEOG) (dependent variable). The study was performed with schools in the following cities (listed alphabetically): Ankara, Adana, Antalya, Ağrı, Aydın, Bartın, Burdur, Bursa, Çanakkale, Elazığ, Gaziantep, Giresun, Hatay, Istanbul, Izmir, Kayseri, Kırşehir, Kocaeli, Konya, Malatya, Nevşehir, Ordu, Osmaniye, Sakarya, Siirt, Şanlıurfa, Tekirdağ, Tokat, Uşak. The cities were such chosen to represent the wide geography and cultural variation within Turkey. The participants were enrolled students in the: 9th, 10th, 11th and 12th high-school grade from 29 different cities geographically spread throughout Turkey during the academic year of 2016–2017. Surveys were conducted electronically with supervision during the period of January 2017 through July 2017. The protocol of delivery was approved from the institutional board as well as the principals of respective schools. Prior to the beginning of the study signed informed consent forms were obtained from all the participating students’ parents.

Our study enrolled a total of 462 students. Inclusion criteria were: to be a Turkish literate student, age 14–17 and enrolled in school in 9th–12th grade level, with a clear health bill (no medical conditions). Out of all 462 students, 386 filled the online form of the survey while 56 preferred to complete the paper form. All surveys were completed in the school setting. Out of 462 students, 54 who filled the online survey (using Google Docs platform) did not complete the survey entirely, while 110 students out of the rest did not fully or adequately answer all the questions on eating habits, resulting in the elimination of their answers from the evaluation. This yielded a final working population of 298 student participants, who provided complete answers to all accounts. Participants were conveniently sampled, while prior relevant mode of sampling experience exists as well as experience with analyzing data from similar studies and settings and for similar participant numbers [7]. We performed power calculations using EHS score (assessing eating habits quality) as the primary outcome. Based on those parameters, a sample-size of 265 participants would be deemed adequate to detect a difference in EHS score.

### 2.2. Scholastic Performance Assessment via TEOG Standardized Examination Scores

Students’ TEOG scores were used as the study’s dependent variable. TEOG is a central examination for admission to senior-high school in Turkey. In each of the two semesters (comprising the academic year) in the Turkish secondary level education system (ages: 12, 13, 14 years old), there are six centrally administered standardized examinations, namely taken in the following subjects: Turkish (language), Mathematics, Science and Technology, Social Studies, English and Religion Culture. Hence, students take 12 centrally administered standardized tests per year, six in each of the two semesters collectively comprising students’ TEOG score for that year. This is done for three consecutive years for 7th, 8th and 9th grade hence ages 12 through 14 years old. Each TEOG score is calculated over 700 maximum points possible. On the TEOG common test score, the mean values calculated over the three grades earned points are averaged to obtain the normalized TEOG placement score for admission to senior-high school. TEOG examinations function as a midpoint of evaluation for students and is used by the Turkish Ministry of Education as a benchmark and quality control checkpoint for the basic education system across the country [12].

### 2.3. Data Collection

In the study presented herein, a survey was used in accordance with the level of understanding of the students. The success of the students was evaluated according to the results of TEOG examinations which are applied throughout Turkey. In this questionnaire, there are questions about students’ sociodemographic and socioeconomic status, exercise status, and meal/diet assessment. In the section where socio-demographic and economic characteristics were assessed, questions included inquiries about age, sex, family type, education level of parents, working status of parents.

**Figure A1:**
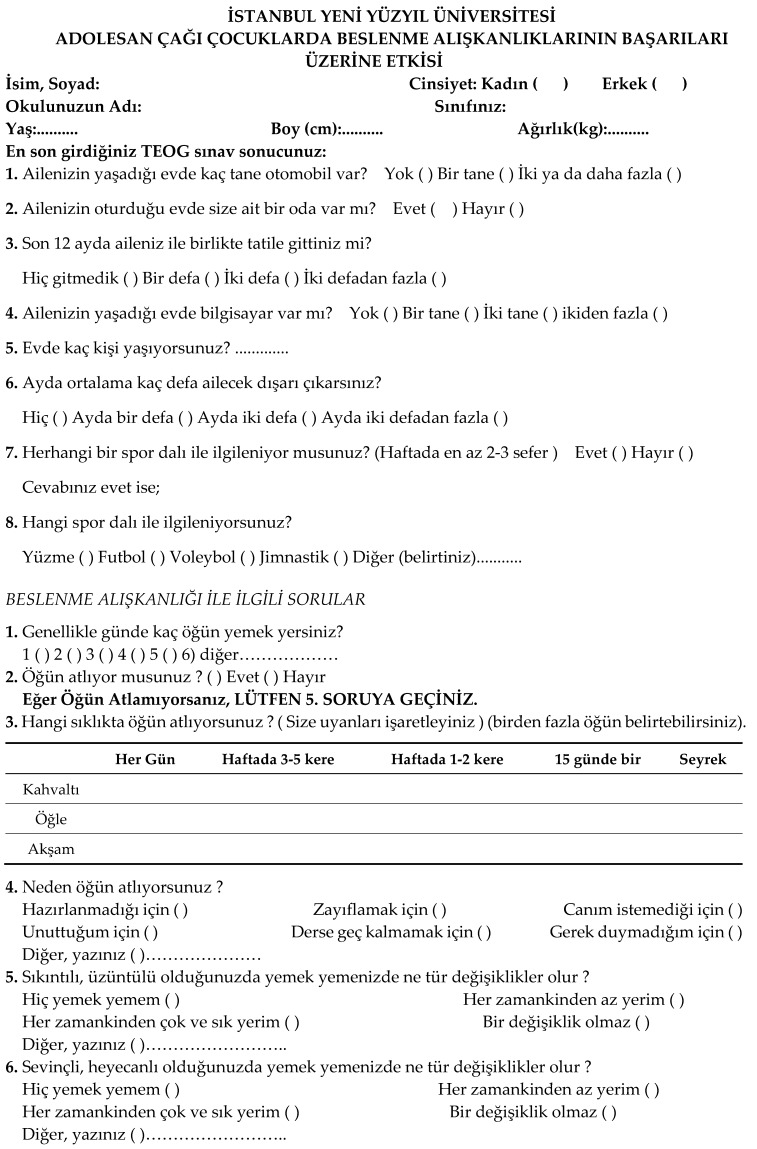

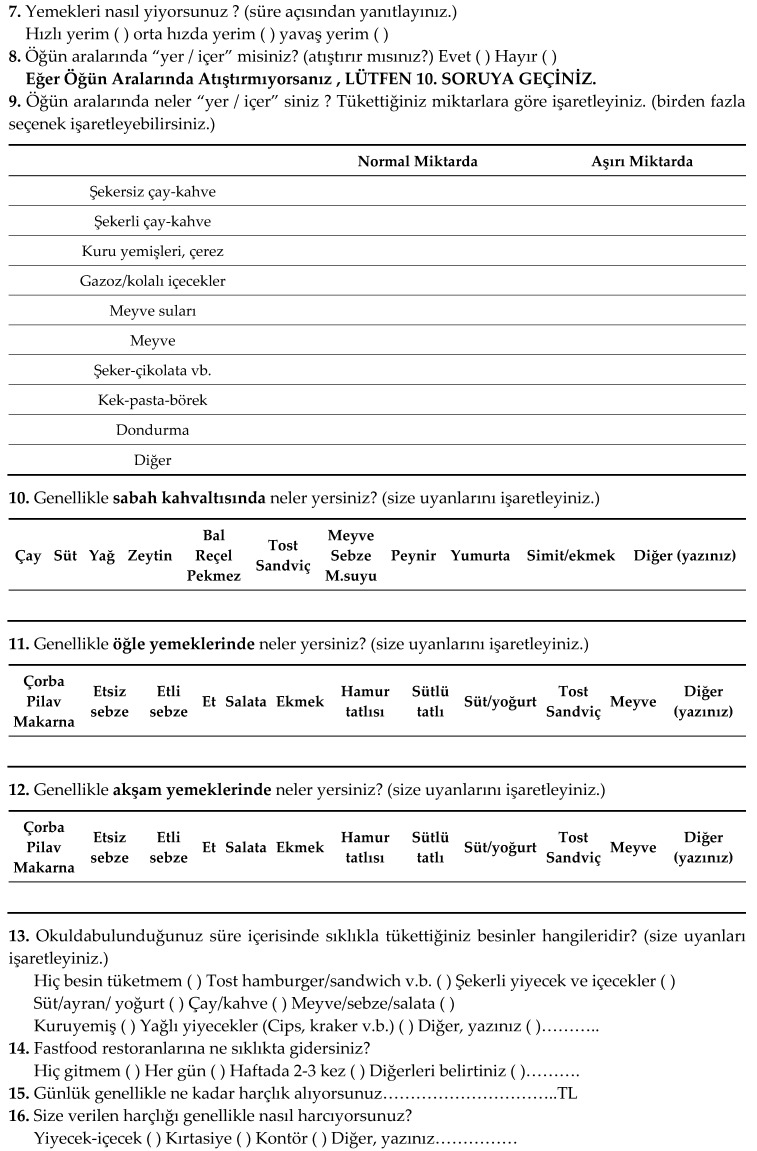
Survey Form Used in Research (Original Version; Turkish).

**Figure A2:**
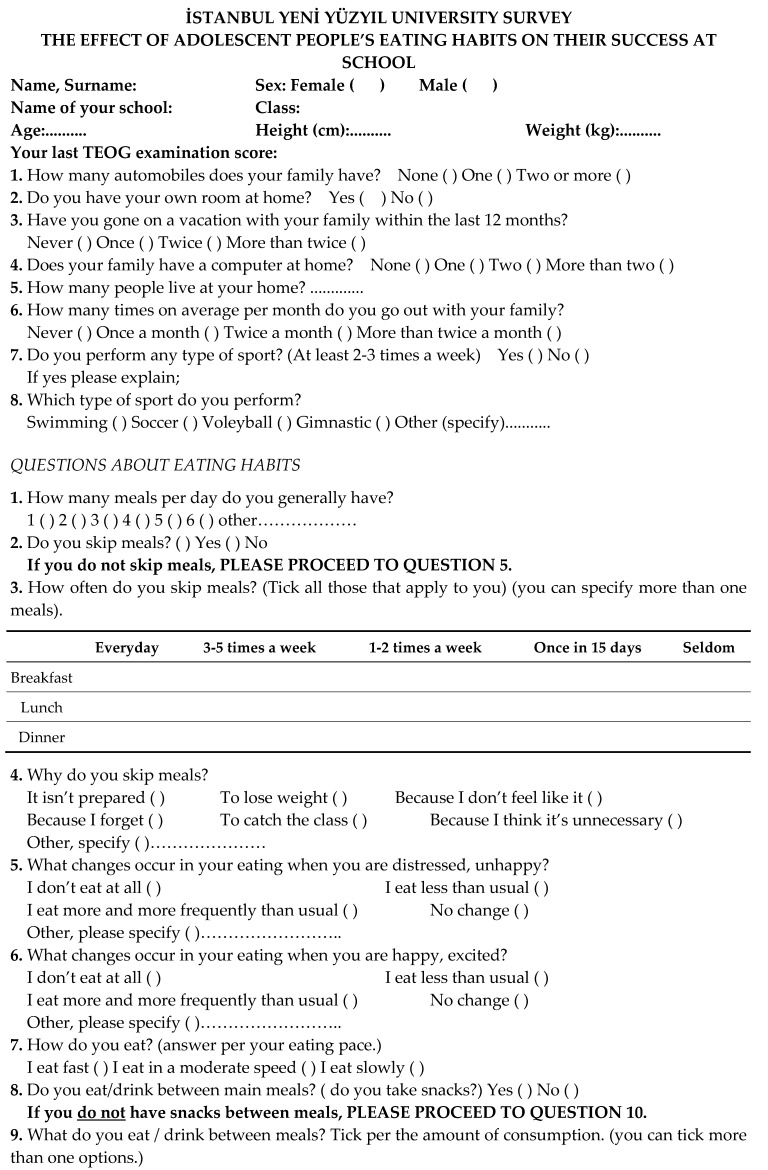

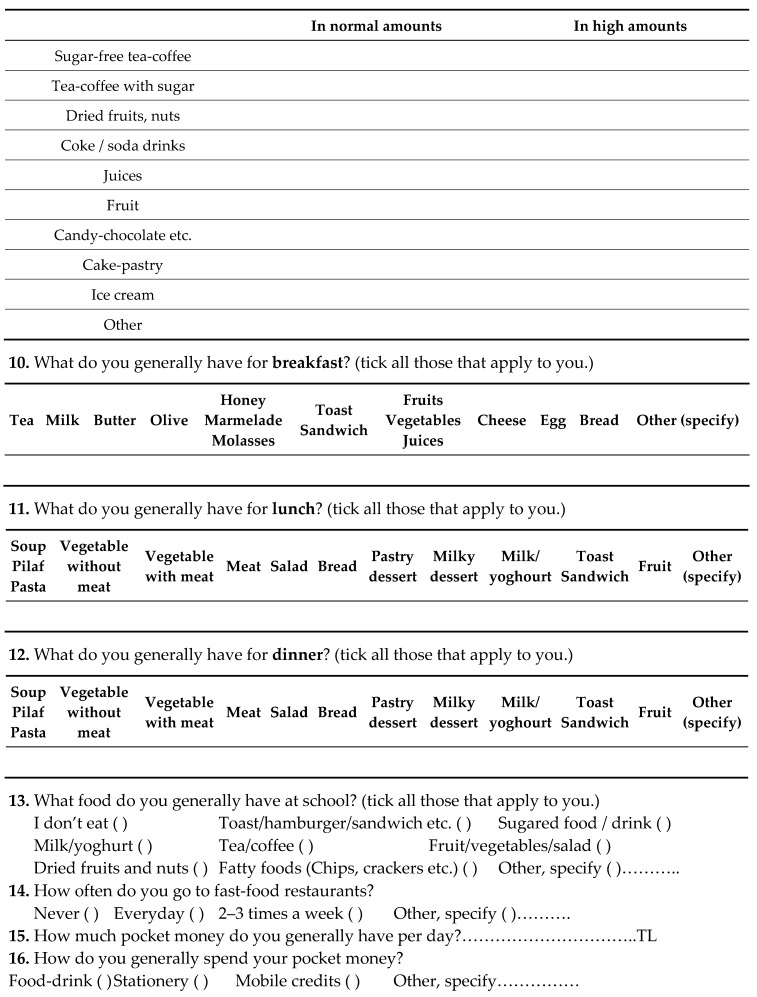
Survey Form Used in Research (English translated version).

Students′ eating habits were evaluated using the eating habits score (EHS). For the questions about EHS and the scoring system, the survey form on “eating habits”, developed and used by Arslan et al. (1994), while also independently used by Alkış (2004) [13,14], was used. The EHS is a scoring system specifically developed for Turkey and validated within the Turkish adolescent population. Total maximum points on the scoring system is 62 when eating habits are evaluated (highest quality of eating habits). Scoring over 48 is classified as good eating habits while scoring in the 35–48 range is considered medium. A score value below 35 denotes poor eating habits from a health standpoint. Furthermore, based on their answers, students were grouped with regards to whether they typically consume a balanced diet according to the food groups consumed during the main meals. Foods are divided into five groups in this evaluation (group 1: meat, eggs, dry legumes; group 2: milk and dairy products; group 3: vegetables and fruits; group 4: cereals and bread; group 5: fats and sugars). The consumption of at least three of them is considered to characterize a balanced diet. The EHS as an assessment tool, provides insight as per the amount and quality of food consumed. The questionnaire included follow-up questions thus allowing the dietitian-analyst to assess the meal-timing and meal-skipping behavior of participants as well as the general types of foods consumed (e.g., sugary/“junk” food). The evaluation of all the dietary data was conducted by the same trained Registered Dietitian Nutritionist licensed in Turkey. The questionnaire used is provided in the appendix section below (Appendix A) as supplemental material both in its original (Turkish) version (Figure A1) and its translated into English version (Figure A2).

### 2.4. Anthropometric Assessments

Height and weight information for the participating students was collected and Body Mass Index (BMI) was calculated according to standard practices. The ideal weight of students was evaluated according to relevant percentile tables. BMI classification was conducted with respect to age and sex. A percentile value <5 was considered significantly underweight, between 5–15 was considered underweight, 15–85 normal weight, 85–95 mildly obese and >95 obese. Student height evaluations were based on height reference values according to sex; while specifically, a percentile <5 is very short, 5–15 is short, 15–85 is normal, 85–95 is tall and >95 is considered very tall. In calculating percentiles, body mass index-for-age percentile tables for boys and girls of age 2–20 years were used [15]. These are standardized classifications and reference points typically used for respective populations in Turkey and are in accordance with World Health Organization (WHO) guidelines [16].

### 2.5. Family Affluence Scale

Socioeconomic status (SES) of student participants was determined using the Family Affluence Scale (FAS), used in the Turkish setting before [17,18]. The FAS system produces classification based on answers provided [17]. Evaluation of SES via FAS scoring into levels/categories is specified as follows: level 1 (FAS score: 0–3) low prosperity, level 2 (FAS score: 4–5) moderate prosperity and level 3 (FAS score 6–7) high prosperity status [17,18].

### 2.6. Ethical Statement

All subjects gave their informed written consent for inclusion before they participated in the study. The study was conducted in accordance with the Declaration of Helsinki, and the protocol was approved by the Ethics Committee of Istanbul Yeni Yuzyil University (project identification code: SBF-120705005-2017).

### 2.7. Statistical Analysis

Participants were conveniently sampled at a level that has been deemed adequate power-wise previously [7]. The statistical analyses were performed using three-way ANOVA test to analyze statistical relations between the survey variables. Interaction between SES, EHS, meal skipping, and TEOG score were assessed.

## 3. Results

### 3.1. General Characteristics of Participants

A total of 298 students, of which 224 were female (75.1%) and 74 male (24.9%), participated in the study. The students’ age spanned from 14 to 17 years, with the majority in the 17 years old age category (41.2%), while student distribution according to school types is primarily from public schools (Table 1). While 240 participants (80.5%) were studying at public schools, 58 (19.5%) were studying at private schools (Table 1). Grade-wise, representation was from the following grade levels: 9th, 10th, 11th and 12th. The highest participation was from 11th grade with 96 participants (32.2%), while the least was from 12th grade with 48 (16.2%) (Table 1). While 111 students (35.2%) are classified as low socioeconomic status (SES) in the survey, 132 students (45.5%) are categorized as middle level, and 55 students (17.3%) are at high level (Table 1). In terms of BMI classifications, a combined 18.2% of participants is categorized as mildly obese or obese, 67.4% as normal while 14.4% as underweight. By this token, about 1/3 of the participants are at a suboptimal weight/height ratio for their age (Table 1). These findings corroborate previous reports/observations [7].

### 3.2. Findings Regarding Students’ Eating Habits

Over half of the students (64.4%) skip at least one meal a day, while 35.6% that do not skip meals. Students often skip their main meals in a variable fashion. The most often skipped meal is breakfast, while the least skipped meal is dinner (Table 2). More specifically, 161 students (54.0%) skip breakfast, 110 students (36.9%) skip lunch, while dinner being the least skipped meal was reported to be skipped by 106 students (35.6%) (Table 2). When evaluating students’ eating habits in terms of how healthy those are, a mere 17.2% scored above 48 points in the EHS signifying healthy eating habits over the last one year. The majority (43.3%) was middle range while a significant 39.5% scored suboptimal (Table 3).

### 3.3. Relationship between Scholastic Performance and: SES, EHS, Meal Skipping

In this study we performed analyses investigating the relationship of SES, EHS and meal skipping practice regarding scholastic performance as measured through standardized test TEOG scores. Our results showed that there is a significant relationship between the socioeconomic status of the families and the scholastic success of the students in terms of TEOG scores (Table 4). More specifically, our results indicate that the lower the SES, the lower the TEOG scores in a dose dependent manner. Furthermore, TEOG scores do follow significantly the EHS as well. More specifically, the higher the EHS the healthier the diet and the higher the TEOG scores, indicating better scholastic performance (Table 4). The higher the frequency of meal skipping, the lower the TEOG scores, suggesting poorer scholastic performance. Meal skipping, in itself, is not necessarily strongly associated with FAS. However, the amount consumed, and the quality of the meal plausibly are more related to FAS, although meals typically consumed by children prior and at school are less sizeable than dinner at home. Skipping a meal in these ages may be more of an issue of play and/or other distractions that avert adolescents’ attention from having a meal. Overall, there appears to be an association between diet and academic performance assuming that children’s dietary patterns remain fairly consistent from early childhood through high-school years, so a better diet may contribute to subsequent better academic performance.

## 4. Discussion

Our work herein aimed to produce information regarding the relationship between quality of dietary habits and scholastic performance in Turkish adolescent students, an understudied and under-referenced topic. This was a pilot study, that assessed the quality of dietary habits through a validated questionnaire specially designed for this population and took into consideration socioeconomic status as determined by the FAS. Scholastic performance was assessed through scores on standardized national centrally administered tests (TEOG) used eventually in part for college admission. Our results did show a significant linear relationship between socioeconomic status and student success, as well as healthy eating and student success independently.

In our work presented here, we observed that as the economic status of the families of student participants improved, it was seen that the level of TEOG scoring, signifying better scholastic performance, also improved significantly. Interestingly, what we also found was that as the SES of families increased so did the EHS, indicating better eating habits for the students coming from higher SES families. The paradigm that higher SES is typically a reflection of higher education and thus higher health consciousness and greater importance placement on health, nutrition and education on the parents’ part is seen. Furthermore, higher SES is typically in accordance with better income thus better access to healthier foods, healthcare (especially of preventive nature and prophylaxis) and overall health status. The combination of interest/importance placement, knowledge and attitude with means and access to better nutrition can support maximally better scholastic performance for students. These relationships have been identified and supported by the findings of other researchers in a variety of settings. In a recent study conducted in Japan, Yamada and collaborators showed that children′s poor lifestyles and low socioeconomic status were significantly associated with low academic performance among Japanese children [19]. In separate studies, also in Japan, Nakahori et al., concluded that a good home environment relates to children′s good dietary behaviors, positive lifestyle factors, and good overall health. The authors concluded that it is important to maintain a good home environment, such as by raising parents′ food awareness, increasing opportunities for communication between children and parents, and having children help with household chores to improve children′s dietary behaviors, lifestyle factors, and health [20].

In another study in China, Li et al., 2018 reported that boys who skipped breakfast and had short night-time sleep duration, ≤6 h/night, were more likely to exhibit poor health status. Similarly, they found that girls who skipped breakfast, and had night-time eating patterns, personal computer use >4 h per day, and short night-time sleep duration, ≤6 h/night, were more likely to exhibit poor health status. Poorer health status predicted lower academic performance in both sexes (sex-independent finding) [21]. Likewise, in our study we did see that there is a significant relationship between students′ success and their status as per meal-skipping, with students skipping meals performing lower in terms of TEOG scores. Recently, Viljakainen and colleagues showed that avoiding fruits and vegetables and following irregular breakfast and dinner patterns were all associated with underweight or excess weight in Finnish adolescents [22]. Hence, their work illustrated that optimal weight achievement is more difficult and less likely among adolescent with poor dietary habits. In a study conducted in Mexico City with students (age: 6–12 years old), Vilchis-Gil and co-workers demonstrated that poorer eating habits as well as less physical activity were associated with higher risk for obesity [23]. In a study conducted in Madrid, Spain with 180 children between 9 and 13 years of age, scholastic aptitude was examined using the scholastic aptitude test (SAT-1) against a 7-day food record. Researchers concluded that students who took adequate breakfast regularly achieved better reasoning scores in the SAT-1 test, a finding indicating a dietary habit-performance relationship even within otherwise well-nourished populations [24]. Moreover, a recent study examined the seventh wave of the Early Childhood Longitudinal Study, Kindergarten Class 1998–99 (ECLS-K) dataset. The eighth-grade year participants’ data, were analyzed, yielding a 9720 data point sample size. Researchers used linear regression analyses to assess the relationship among nutrition, physical activity, and academic achievement, while controlling for socioeconomic status, age, and sex [25,26]. In Norway, researchers demonstrated that regular meal pattern, healthy food consumption and physical activity were all associated with increased odds of high academic achievement in Norwegian adolescents. On the contrary, consumption of unhealthy foods and beverages, was associated with decreased odds of high academic achievement [27]. Researchers in the US concluded that non-active, unhealthy nutrition, participants scored consistently lower for reading, math, and science standardized tests when compared to those classified as active, healthy nutrition regardless of sex [25,26]. Furthermore, research in Australia focusing on secondary analysis to examine associations between a range of dietary behaviors and children′s academic achievement, revealed that dietary behaviors are associated with higher academic achievement [28]. In Pakistan, Mushtaq et al., demonstrated that dietary behaviors, physical activity and sedentary lifestyle all constitute independent predictors of overweight and higher BMI among Pakistani primary school children and are significantly affected by the child′s socio-demographic characteristics [29]. All the aforementioned observations and conclusions are in accordance with our obtained results and derived conclusions.

Regarding breakfast status in particular, there have been numerous studies suggesting that breakfast skipping is negatively associated with scholastic performance. Most interestingly, a meta-analysis review by Hoyland and colleagues, reviewing several high-quality studies on breakfast consumption versus no breakfast intake demonstrated, positive outcomes from eating breakfast, particularly as these relate to highly demanding tasks and most importantly especially for children who are considered more vulnerable (e.g., lower SES, lower IQ, lower overall health status and poorer diet) [30]. Similarly, we found a strong negative relationship between breakfast skipping practice and scholastic performance as assessed via TEOG scores. Furthermore, breakfast skipping may influence more strongly the lower SES students given the potential additive negative effect of the two conditions. In this sense, optimal dietary practices may benefit more the more vulnerable adolescent students in terms of their scholastic performance. In fact, recently conducted empirical studies demonstrated a significant, negative association between nutrition intake and learning among children from low-income families. Specifically, disadvantaged children with less healthy diets demonstrate lower math skills at kindergarten entry, a trend that usually holds-up in later years in the educational system [31]. An extensive meta-analysis in Korea studying adolescents, investigated adjusted odds ratios (AORs) of dietary habits for school performance using multinomial logistic regression analyses with complex sampling. The results obtained, confirmed findings from previous studies regarding school performance and dietary patterns showing a positive association with eating breakfast and consuming fruits and milk versus a negative association with soft drinks, instant noodles, fast food, and confections [10].

In a recent comprehensive review of the evidence issued by Cochrane Reviews, it was concluded that school-based dietary interventions may benefit general school achievement in children with obesity particularly, although the entire student population would benefit at least marginally [32] with, arguably, lasting results. In fact, adherence to a Mediterranean Diet (MD) was shown to be positively related with better academic success in college students. While a review of studies documented that poorer students′ health status was associated with lower MD adherence while inversely higher MD adherence was correlated with lower depression risk, whereas higher perceived stress score with lower fruit and vegetables intake [33]. Fruits and vegetables in particular, have been associated with a plethora of benefits attributed to their surplus of a rather wide variety of bioactive compounds [33,34].

Studies at various settings in Turkey [35,36] have identified that poor academic performance is related to lower SES as well as poor dietary habits, hence corroborating our findings further. More specifically, research conducted in Turkey by Duman, 2012 and Diremler, 2009 [36,37] independently reported that students skipping meals statistically performed consistently lower in school than those who did not skip meals, thus led to similar conclusions to ours. More expressly, Duman’s work associated EHS with scholastic performance in the same way that our results suggest. In a study by Meydanlıoğlu, it was observed that students with healthy food intake are more successful than students who consume sugary foods, salty snacks or fast food in general [9]. Additionally, Ardic and Esin reported that significant predictors of healthy lifestyle behaviors of adolescents, included good relationships with family and friends, having a father who was a college graduate, and good health perception, thus indicating that SES is a predictor of good health and better dietary practices [38]. Furthermore, work by Ardic and Erdoğan showed that adolescents in relevant intervention groups showed improvements in nutritional behavior, physical activity and stress management [39]. These improvements concerned increased walking, measured in number of physical steps taken per week, daily fruit and vegetable consumption, and the daily water consumption. While their nutrition/physical activity related knowledge significantly increased, their weight and anxiety symptoms significantly decreased. The consensus in the nutrition community seems to support that while packaged foods, fast food, sugary and fatty foods all tend to possibly affect brain development and hence scholastic aptitude negatively, other food groups such as vegetables, fruits, fish, dairy products are preferred, in terms of supporting children′s capacity for greater success in school. In a dynamic food scene with various challenges especially in terms of child nutrition in a country in epidemiological transition, it is important to establish a series of policies as per the tools that can improve knowledge and attitude of children towards nutrition, as a supportive element to their academic success and beyond [40]. While knowledge does not guarantee positive behavioral change, it is however a first critically important step of empowerment and enablement, necessary towards a positive change.

Overall, the literature suggests that good regular dietary habits are the best way to ensure optimal mental and behavioral performance at all times. However, it remains challenging as per the way to attain a better nutrition/dietary set of habits and practices even more so in the adolescent student population in general, and Turkey in particular. In order for positive behavioral modification to be successful, knowledge transfer (i.e., educational modules for students and parents) while important, may not be efficient enough. Motivational interviews and relevant practices as well as participatory actions should maybe also be explored for better and sustained results. An interesting example of such was the practice of school garden development. In an encompassing study that reviewed a series of school-day garden interventions with measures of dietary and/or academic outcomes the results were rather interesting. The analyses concluded that garden-based learning does not negatively impact academic performance or fruit and vegetable consumption and may favorably impact both. Furthermore, the overall dietary habits of students as well as academic performance seem to improve [41]. Experiential learning thus seems to be supportive towards a positive behavioral change as per dietary habits, in turn positively affecting overall health and academic performance in adolescent students. More research needs to be conducted along the lines of potential interventions targeting the improvement of nutrition and academic performance in manners that overcome socioeconomic status disparity related barriers and maximize engagement and participation of all interested parties. Limitations of the current study include convenient mode of sampling participants and uneven representation of female versus male participating students. As per the sampling method it is not likely to influence heavily the results since there was a notable geographic distribution and element of randomization in the student population. Regarding the female prevalence in the participating population, it is conceivable that the sex-uneven distribution may bias the results, however given the fact that the main drivers (i.e., SES, demographics) are not differently present the possibility for significant bias is expected to be rather low. In summary, sex is a much less influencing driver and determinant of EHS compared to other variables such as SES.

## 5. Conclusions

According to our work discussed above, there is a positive relationship between adolescent eating habits and their success at school among adolescent students in Turkey. Students with better eating habits as assessed by EHS, were consistently achieving higher scholastic performance as assessed by standardized test scores (TEOG) than those who demonstrate lower EHS. As the quality of nutrition improved, the measured scholastic student success increased as well. Our results corroborate findings of other studies in different countries and settings as well as very few conducted in Turkey. While it should be emphasized that student eating habits may extend significant impact on scholastic successes, more research is needed to identify strategies to induce a positive behavioral change in students as per their nutrition and/or overall lifestyle habits, involving the school setting and families.

## Figures and Tables

**Table 1:** Student Distribution by: sex, age, school type/class and socioeconomic status.

	n	%
Sex		
Female	224	75.1
Male	74	24.9
Age (years)		
- 14	17	5.7
- 15	60	20.1
- 16	97	32.5
- 17	123	41.2
- 18	1	0.5
School Type		
Public	240	80.5
Private	58	19.5
Grade (Level)		
- 9	77	25.8
- 10	77	25.8
- 11	96	32.2
- 12	48	16.2
Socioeconomic Status ^‡^		
Low	111	37.2
Middle	132	45.5
High	55	17.3
Body Mass Index (BMI) ^Υ^		
Percentile < 5th	17	5.7
5th ≤ Percentile < 15th	26	8.7
15th ≤ Percentile < 85th	201	67.4
85th ≤ Percentile < 95th	41	14.1
Percentile ≥ 95th	13	4.1
Total Participants: n = 298 (224 female, 74 male).		

^‡^ Socioeconomic status is evaluated according to the family affluence scale (FAS), family affluence scale analysis; low: 0–3, medium: 4–5, high: >6. ^Υ^ Body mass index (BMI) is categorized according to appropriate percentiles (WHO).

**Table 2:** Student distribution by meal type skipped: breakfast, lunch, dinner.

	n	%
Skipping Meals		
Yes	192	64.4
No	106	35.6
Skipping Breakfast		
Yes	161	54.0
No	136	46.0
Skipping Lunch		
Yes	110	36.9
No	188	63.1
Skipping Dinner		
Yes	106	35.6
No	192	64.4
Total Participants: n = 298 (224 female, 74 male).		

**Table 3:** Eating habits score distribution.

	n	%
Eating Habits Score		
<35	118	39.5
35–48	129	43.3
>48	51	17.2
Total Participants: n = 298 (224 female, 74 male).		

**Table 4:** Assessment of students′ success via TEOG scores relative to FAS, EHS and meal skipping status.

Family Affluence Scale ^♦^	N	TEOG Score: $\bar{x}$ ± SEM
Low	111	358.8 ± 6.8
Medium	132	391.2 ± 6.0
High	55	401.2 ± 9.4
		***P* = 0.0001**
**Eating Habits Score**	**N**	**TEOG Score:** $\bar{x}$ **± SEM**
<35	118	363.2 ± 7.2
35–48	129	386.8 ± 6.1
>48	51	401.5 ± 9.0
		***P* = 0.003**
**Meal Skipping**	**N**	**TEOG Score:** $\bar{x}$ **± SEM**
Yes	192	372.1 ± 5.1
No	106	398.5 ± 7.2
		***P* = 0.003**
Total Participants: n = 298 (224 female, 74 male).		

♦ Family affluence scale; low: 0–3, medium: 4–5, high: >6. TEOG values are mean scores ± SEM. Statistical significance was accepted at: *P* < 0.05 (denoted in bold).
